# Plant Molecular Farming – Integration and Exploitation of Side Streams to Achieve Sustainable Biomanufacturing

**DOI:** 10.3389/fpls.2018.01893

**Published:** 2019-01-18

**Authors:** Johannes F. Buyel

**Affiliations:** ^1^Fraunhofer Institute for Molecular Biology and Applied Ecology IME, Aachen, Germany; ^2^Institute for Molecular Biotechnology, RWTH Aachen University, Aachen, Germany

**Keywords:** biomass conversion, biopharmaceuticals, biorefinery, molecular farming, plant secondary metabolites, process sustainability

## Abstract

Plants have unique advantages over other systems such as mammalian cells for the production of valuable small molecules and proteins. The benefits cited most often include safety due to the absence of replicating human pathogens, simplicity because sterility is not required during production, scalability due to the potential for open-field cultivation with transgenic plants, and the speed of transient expression potentially providing gram quantities of product in less than 4 weeks. Initially there were also significant drawbacks, such as the need to clarify feed streams with a high particle burden and the large quantities of host cell proteins, but efficient clarification is now readily achieved. Several additional advantages have also emerged reflecting the fact that plants are essentially biodegradable, single-use bioreactors. This article will focus on the exploitation of this concept for the production of biopharmaceutical proteins, thus improving overall process economics. Specifically, we will discuss the single-use properties of plants, the sustainability of the production platform, and the commercial potential of different biomass side streams. We find that incorporating these side streams through rational process integration has the potential to more than double the revenue that can currently be achieved using plant-based production systems.

## Introduction – Benefits and Drawbacks of Plant Molecular Farming

A vast body of literature has accumulated describing the potential of bio-based strategies for sustainable manufacturing, the production of biofuels, and the reduction of carbon dioxide emissions. This article explores how the different aspects of a sustainable bio-industry can be integrated into plant-based processes for the production of recombinant proteins, and how this affects overall process economics.

### First-Generation Advantages of Plant-Based Expression Systems

**FIGURE 1:**
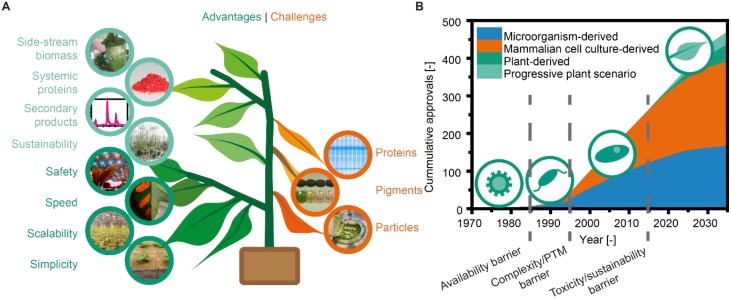
Overview of plant-based expression systems. **(A)** Advantages and challenges typically encountered during plant molecular farming. This study expands the previously reported aspects (dark green and orange) with new potential benefits (light green). **(B)** Approvals of recombinant-protein-based biopharmaceuticals. Starting in 1985, the approved drugs expressed in microorganisms (prokaryotic and eukaryotic), mammalian cells, or plant cells are plotted in 5-year intervals based on data published up to 2014 (Walsh, 2014). The forecast up to 2035 is based on reported product pipelines (e.g., Gleba et al., 2014).

Shortly after the first successful expression of recombinant antibodies in plants (Hiatt et al., 1989), it became apparent that plants offer several potential benefits compared to more established platforms such as mammalian cell cultures (Figure 1A). First, plants are inherently safe because no human pathogens replicate in plants, resulting in a low pathogen load and a low risk of process-related contamination (Commandeur et al., 2003). Second, the cultivation of plants is simple because there is no need for a sterile environment: intact plants can rely on their native immunity to keep pathogens at bay (Sack et al., 2015). Furthermore, inexpensive (∼0.002 € L^-1^), defined fertilizer solutions are sufficient for cultivation (Buyel and Fischer, 2012) as opposed to the expensive media required for mammalian cell cultures, which often cost more than 50 € L^-1^ (Xu et al., 2016). In this context, the third advantage is that cultivation of transgenic plants in particular can, in theory, be expanded to the agricultural scale, i.e., several thousand hectares. In combination with biomass yields of ∼100,000 kg ha^-1^ y^-1^ for tobacco (*Nicotiana tabacum*) (Stoger et al., 2002) and expression levels of up to 2 g kg^-1^ (Zischewski et al., 2015), this can easily facilitate the production of recombinant proteins at the multi-tonne scale, e.g., 100 kg ha^-1^ y^-1^ assuming an overall process recovery of 50%. Even under more controlled conditions as found in a greenhouse (Ma et al., 2015; Sack et al., 2015) or vertical farm (Wirz et al., 2012; Holtz et al., 2015), multi-tonne scale production may still be possible (Buyel et al., 2017). A fourth advantage is that recombinant protein expression in plants can be achieved ∼8 weeks after receiving the corresponding DNA sequence (Shoji et al., 2012), typically using transient expression mediated by infiltration with *Agrobacterium tumefaciens* and/or viral vectors (Peyret and Lomonossoff, 2013; Gleba et al., 2014). This rapid production allows quick responses to epidemic or pandemic threats, as impressively shown by the production of an anti-Ebola antibody cocktail (Qiu et al., 2014).

Additionally, plants can perform the same post-translational modifications as mammalian cells (Strasser, 2016) which means they are superior to prokaryotic systems for the expression of complex proteins such as mAbs or membrane proteins (He et al., 2014). Even though plant-specific glycosylation can enhance the pharmacologic activity of certain products (Grabowski et al., 2014), several transgenic plants and plant cell cultures have been established that allow the recombinant protein to receive an authentic human glycosylation pattern (Strasser et al., 2008; Hanania et al., 2017; Mercx et al., 2017), reducing any likelihood of immunogenicity, however remote (Shaaltiel and Tekoah, 2016). Plants can also be used to produce intrinsically disordered proteins (Gengenbach et al., 2018b) which are naturally abundant in plants (Covarrubias et al., 2017) or to facilitate the manufacturing of products like viscumin (Gengenbach et al., 2018a), a lectin with anti-cancer activity (Zwierzina et al., 2011). These cannot be synthesized efficiently in mammalian cells or prokaryotes due, respectively, to their toxicity and complex structure (Gengenbach et al., 2018a).

### Mitigation of Initial Drawbacks

Despite the advantages of plants for upstream production, the most challenging and cost-intensive aspect of recombinant protein production in plants has been DSP, accounting for up to 80% of the total process costs (Wilken and Nikolov, 2012; Buyel, 2015). The major cost-drivers during DSP were (i) the high particle burden of primary extracts, requiring extensive clarification, (ii) the large amounts of HCP impurities which had to be separated from the product, and (iii) the presence of plant secondary metabolites, including pigments and phenols, that might permanently bind to, and thus alter, the product. Particles, HCPs, and metabolites are typically released due to the thorough homogenization required to extract the product from plant tissues (Buyel and Fischer, 2014e; Hassan et al., 2014). If a recombinant protein is produced in seeds, e.g., to improve product stability and storage properties (Hofbauer and Stoger, 2013), the release of starches during extraction can trigger gel-formation, which may hinders clarification. However, alternative extraction methods such as centrifugal extraction (McCormick et al., 1999) or screw-presses (Buyel and Fischer, 2014b) are now available that can reduce the HCP content and particle burden of primary extracts. Furthermore, effective clarification methods (Buyel et al., 2015b), as well as supporting technologies such as flocculation, filter aids, and pre-coat filtration techniques (Buyel et al., 2014b), have recently been adapted for plant-based systems, reducing the associated costs by more than 75%. Treatment at moderate temperatures (∼65°C), at low pH (∼5.5), or by ultrafiltration/diafiltration (100–300 kDa) can reduce the HCP content in extracts by more than 90%, facilitating product purification and reducing production costs (Hassan et al., 2008; Lightfoot et al., 2008; Buyel et al., 2014a).

Also, extraction-free recovery of recombinant proteins from plants via rhizosecretion has been reported recently for antibodies and other therapeutically active proteins (Madeira et al., 2016a,b). Apart from a reduced HCP and particle burden, this technology is well in line with a shift toward continuous operation seen in the biopharmaceutical industry (Klutz et al., 2015) and compatible with hydroponic plant cultivation. However, the approach is currently limited to transgenic plants as well as products that can be subjected to the secretory pathway and are able to withstand the conditions in the fertilizer/hydroponic solution they are secreted to. Exposing a product to such typically non-sterile and potentially poorly defined conditions may raise regulatory scrutiny.

Expression levels in plants have increased substantially from 0.02 to 0.10 mg kg^-1^ to a current maximum of about 2 g kg^-1^ (Bendandi et al., 2010; Zischewski et al., 2015). Assuming that 1 L of fermentation volume is roughly equivalent to 1 kg of plant biomass, this implies that the productivity of plant-based systems is now in the same order of magnitude as Chinese hamster ovary cells (∼5 g L^-1^) (Kelley, 2007). With an overall yield of 70% for purified mAbs (Ma et al., 2015), and average market prices of 9,200 € g^-1^ in 2008 (Kelley, 2009), this corresponds to a product market value of ∼13,000 € kg^-1^ plant biomass which has to be offset against the production costs that can be 150–250 € kg^-1^. These costs typically include ∼50 € kg^-1^ biomass for upstream production assuming that 150 g green biomass can be harvested per plant (Buyel and Fischer, 2012, 2014a) and ∼200 € kg^-1^ for DSP (Buyel and Fischer, 2012). The latter may reduce to ∼100 € kg^-1^ if advanced clarification techniques including flocculants and filter aids are used to reduce consumables costs (Buyel and Fischer, 2014c; Buyel et al., 2014b).

For leafy expression systems, and in combination with systematic empirical and model-based approaches for a rational DSP design (Buyel et al., 2013; Buyel and Fischer, 2014d), these developments now allow the production and purification of recombinant proteins from plants with an effort comparable to that required for cultured mammalian cells. The latter typically do not require homogenization of the cells as the product is often purified directly from the culture supernatant (Xu et al., 2017). Furthermore, if minimal processing is possible (i.e., relaxed purity requirements apply) (Rosenberg et al., 2015), plants can already compete with other systems according to a number of techno-economic analysis reports (Sharma and Sharma, 2009; Buyel and Fischer, 2012; Tuse et al., 2014; Walwyn et al., 2015; Nandi et al., 2016). In this context, several biological approaches, mostly based on specific fusion partners, have been developed that can facilitate a lean and thus cost-effective product purification strategy. For example, oleosins direct a product to oil bodies in seeds (Laibach et al., 2015), a zein-tag can result in product accumulation in zein storage organelles (Hofbauer et al., 2016).

The initial absence of regulatory guidance has also been overcome by providing a framework for the industry, especially for biopharmaceutical manufacturing, which should further mitigate uncertainty and thus eliminate the investment risks previously associated specifically with plant-based expression systems (Fischer et al., 2012, 2014).

### Remaining Entry Barriers

Despite the promising developments discussed above, major biopharmaceutical companies have not adopted plant-based expression systems even following the approval of the first plant-derived pharmaceutical protein product approved for human use (Mor, 2015). This inertia may reflect the completed depreciation of established fermentation capacities, the constantly increasing product titers reported for mammalian cell cultures [now regularly reaching 5 g L^-1^ or more for mAbs (Rader and Langer, 2015; Xu et al., 2017)], and the advent of increasingly sophisticated single-use technologies which allow flexible process layouts (Gottschalk, 2009). We speculate that once a new class of biopharmaceutical product enters the market and pushes the existing production systems beyond their limits, a substantial shift toward plant-based expression systems may occur. This has a historical precedent: prior to the recombinant DNA era proteins were isolated from natural sources limiting the bandwidth of available products. This was overcome with the advent of recombinant DNA technologies leading to microbial fermentation processes that facilitate the production even of artificial proteins. However, microbes are unable to produce more complex proteins, so that mammalian cell cultures gained immense popularity once the potential of such complex proteins like mAbs became apparent (Figure 1B).

Even though the boundary conditions currently favor established and yet growing fermentation-based production systems for recombinant biopharmaceutical proteins (Ecker et al., 2015), these conditions are likely to change in the mid to long term, once the mAb market faces saturation effects and novel products that are difficult to express in mammalian cells and microbes due to a complex structure and inherent toxicity, gain interest. An increasing demand for sustainable bioprocesses may accelerate this transformation. In the following section, we will provide details and examples for the sustainability of plant-based recombinant protein expression systems and the use of biomass side streams from such systems whereas in the subsequent section, we will apply these thoughts to an existing production process for a recombinant mAb derived from plants to highlight the potential impact on process economics.

## Developing the Potential of Plant-Based Production Systems

### Reduced Investment and Cleaning Validation

Plants grow from seeds during the production process and hence require little pre-installed infrastructure, which can be costly if prices for construction materials increase or are subject to substantial fluctuations. For example, the cost of stainless steel (US cold-rolled coil) increased from ∼0.45 € kg^-1^ in December 2015 to 0.75 € kg^-1^ in April 2017 (de Frutos, 2017) which can have a direct impact on the costs for conventional bioreactors and other multi-use equipment and piping. Additionally, upcoming trade regulations and taxes that have been announced in recent months can increase the prices and affect their volatility. A recent analysis of the capital expenditure to build 60,000 L production capacity for mAbs indicated that single-use production can reduce the investment from ∼300 to 220 million € compared to a stainless steel process (Jacquemart et al., 2016). Other authors have reported similar estimates (Pollard and Pralong, 2018). In contrast, a greenhouse production facility for 250 kg plant biomass output (∼320 m^2^ area) has been built for 500,000 € (∼1500 € m^-2^; including the building and all installations allowing an operation at safety level S1). If scaled to the process size of 60,000 L above and assuming that 1 kg of plant biomass is equivalent to 1 L of fermentation volume, this corresponds to a 120 million € investment for a manufacturing greenhouse and is about half the price for fermentation-based processes. Also, for such an 80,000 m^2^ facility, benefits of scale like improved utilization of media supply and control infrastructure are expected to reduce the building costs to ∼750 € m^2^, decreasing the investment to 60 million € or ∼25% of that for a bioreactor process.

The product quantity and quality obtained from greenhouse cultivations may vary with the changing seasons (Sack et al., 2015) and the infrastructure may provide only limited protection against trespassers. Therefore, fully enclosed and partially automated facilities housing several layers of plant cultivation area, typically referred to as “vertical farms,” have recently been commissioned (Wirz et al., 2012; Holtz et al., 2015). The authors claim that the facilities are cheaper than conventional fermentation-based counterparts and that bulk protein costs for a flu subunit vaccine are 2,000 € g^-1^, respectively. A reliable cost estimate for vertical farming infrastructure may be difficult at the moment because (i) a full cost analysis for such a facility is currently not publically available to our knowledge and (ii) the equipment is often of custom design increasing the engineering costs compared to standard devices used in most fermentation processes. However, information from commercial vendors of vertical farms^1^ are now available and indicate that investment costs for closed, automated cultivation are ∼17.5 € and 18.9 € per kilogram of annual biomass production capacity for a stationary- or a container-based facility with total investments of ∼10.5 million € (600,000 kg biomass y^-1^) and 0.1 million € (6,000 kg biomass y^-1^), respectively. These facilities currently do not cover extraction and DSP equipment, so the investment costs for a fully functional production unit will be higher. However, even if the costs for the additional equipment are of the same magnitude, the total facility costs would only be a small fraction (<10%) of those for a fermentation-based process.

An expected energy price increase of 20% in the next 10 years (Anonymous, 2017) may increase the operating costs for stainless steel equipment as well because energy-demanding steam-based cleaning in place and sterilization in place procedures often used. Furthermore, the production of pharma-grade stainless steel is energy intensive as well requiring 1.8–3.2 MJ per kg steel for the furnace (Kirschen et al., 2009) and 19.2 MJ per kg of final product (Anonymous, 2014), which also increases the carbon dioxide footprint of the corresponding products.

In contrast to stainless-steel fermenters but like single-use bioreactors, an individual plant is only used once in its lifetime for the production of a specific product and is typically completely disintegrated during the extraction process, e.g., using blade-based homogenizers (Buyel and Fischer, 2014e; Hassan et al., 2014). Therefore, without any additional investment or increased consumables costs, plants minimize the risk of product cross contamination during the upstream production phase of a process. In contrast to multi-use fermenters that will likely be used for multiple products, this reduces cleaning and validation procedures as well as the associated effort and costs to near zero (EMEA, 2000; Medina, 2003). The inherent single-use characteristics of plants can therefore directly reduce production costs, as shown for the integration of other single-use technologies with mammalian cell cultures, achieving cost savings of more than 10 million euros at production scale (Barnoon and Bader, 2008; Levine et al., 2013; Rogge et al., 2015). However, licensing fees as seen for special mammalian production cell lines such as CHO DG44 (Reinhart et al., 2015) may also apply to plant lines, e.g., those offering humanized glycosylation (Jansing et al., 2018).

### Decoupling Manufacturing From Resources of Limited Availability or Volatile Costs

As plant bioreactors grow anew in each production cycle (an analogous concept in traditional processes would require self-assembling bioreactors), their single-use properties are not subject to markups that can arise due to fluctuations in supply and demand, or shortages of raw materials. For example, even though a forecast can be difficult (Bernard et al., 2017), the depletion of fossil resources along with other factors may drive the price of crude oil from $48 per barrel (∼0.27 € L^-1^) to $150 per barrel (∼0.82 € L^-1^) by 2040, for an intermediate price increase scenario (Conti et al., 2016). This will have a direct impact on diverse plastics and organic chemicals (Weinhagen, 2006), potentially increasing prices by more than 100%. In turn, the raw materials costs could translate into substantially higher prices for single-use process equipment, increasing the cost of goods for processes relying on such equipment, and potentially reducing the economic feasibility of the process. Single-use technologies may also be subject to additional cost inflation, e.g., due to penalties for processes with a large ecological footprint based on political decisions to reach goals for environmental and climate protection (Victor and Leape, 2015; Rogelj et al., 2016, 2017). These decisions would increase costs for waste disposal and there may be specific fees for processes with a large carbon dioxide footprint (Smith et al., 2001). Therefore, incorporating biodegradable materials or carbon dioxide fixation strategies can be promising attempts and will be discussed in the next two sections.

### Biodegradable Plant-Based Expression Systems

Replacing fossil resources with bio-based bulk chemicals may limit the price increase for plastics (Wilke, 1999; Hermann and Patel, 2007), but it is unclear when the prices will stabilize (Nordhoff et al., 2007). Still, a number of biodegradable plastics have been developed, including polylactic acids, polyhydroxyalkanoates, regenerated cellulose, and thermoplastic starch (Lopez et al., 2015; Rujnic-Sokele and Pilipovic, 2017). These could be used to manufacture single-use equipment and thus reduce the ecological footprint of cell culture-based as well as plant-based processes. However, some of these novel materials do not decompose in soil and others have turnover times of more than 6 months (Kyrikou and Briassoulis, 2007; Mohee and Unmar, 2007), as observed for pro-oxidant infused polyethylene films and MaterBi resin, respectively (Briassoulis and Dejean, 2010; Novamont, 2015). Degradation can be accelerated if composting is carried out under optimized conditions (Briassoulis and Dejean, 2010), e.g., after mechanical pre-treatment (Kyrikou and Briassoulis, 2007), but this will require special facilities for degradation which again increases recycling costs. In addition, not all biodegradable plastics are suitable for single-use applications, especially in highly regulated environments such as the production of biopharmaceuticals. This reflects strict norms regarding stability, leachables and extractables, particularly during upstream production in cell culture-based processes where contact times of several days are common (Kyrikou and Briassoulis, 2007; Briassoulis and Dejean, 2010). It is therefore prudent to exploit the inherently sustainable single-use potential of expression systems like plants where the greatest advantage is found during upstream production because DSP is now similar to conventional cell culture and fermentation processes, i.e., it relies to a large extent on single-use equipment such as filters for clarification.

**FIGURE 2:**
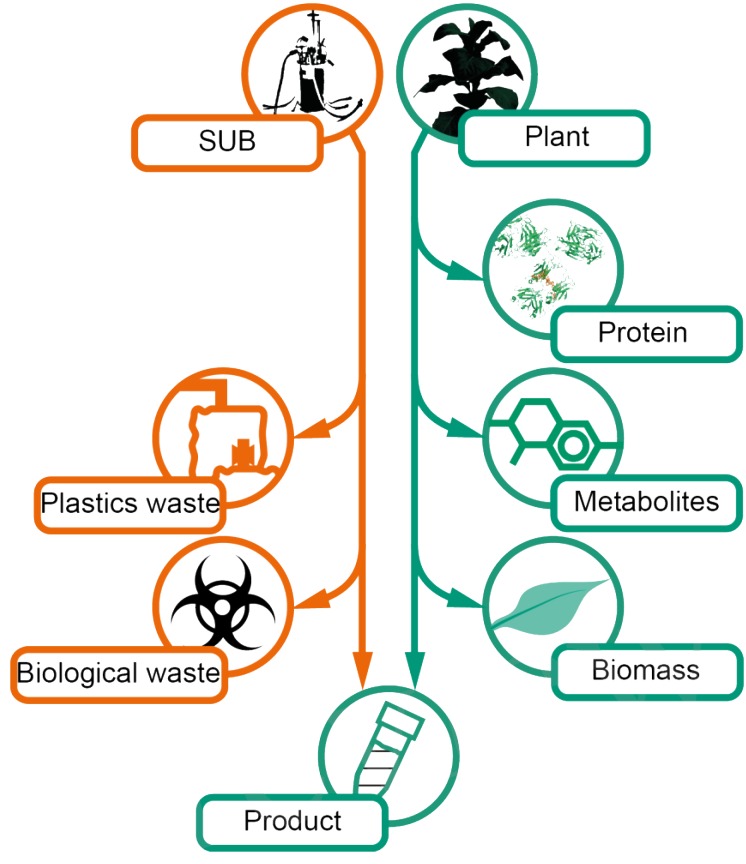
Comparison of waste streams generated in a mammalian cell culture-based process relying on single-use equipment and a plant-based counterpart. SUB, single-use bioreactor.

For plants, sustainability is clearly promoted by the fact that all plant material remaining after product extraction is rapidly biodegraded under typical environmental conditions, ultimately forming basic organic and inorganic substances due to the activities of nematodes, fungi, and bacteria (Miki et al., 2010; Zhu et al., 2013; Ebeling et al., 2014; Baldrian, 2017). This helps to reduce the amount of waste requiring expensive and dedicated disposal facilities, as is the case for mammalian cell cultures. For example, it is necessary to dispose ∼61 kg of plastics (mainly polypropylene) per batch for a 1,000-L single-use cell culture process based on list of consumables corrected for the life cycles (Rawlings and Pora, 2009; Figure 2). This waste material may require heat inactivation to at least 60°C before disposal (Gregoriades et al., 2003). The energy costs are currently 0.024 € MJ^-1^ (Boulamanti and Moya, 2017). In combination with a heat capacity of 4,186 J K^-1^ kg^-1^ for the cell culture fluid and ∼1,500 J K^-1^ kg^-1^ for the plastics components (Fujino and Honda, 2009) as well as costs for solid and liquid waste disposal of ∼0.1 € kg^-1^ and 0.002 € L^-1^, respectively (Rodriguez-Garcia et al., 2011; Horrnweg and Bhada-Tata, 2012; Hernandez-Sancho et al., 2015), this can account for costs of 0.15 € L^-1^ fermenter volume and batch (0.05 € for energy and 0.10 € for waste).

In contrast, the liquid waste in plant-based processes is minimized because plant growth promoting fertilizer solutions can easily be used for several production batches as component concentrations (e.g., trace elements or nitrogen source) can be individually re-adjusted and a sterile handling is not required. Furthermore, heat inactivation of liquid and solid wastes is not required if transgenic, non-infiltrated plants are used for production because the plant homogenate generated during product extraction does not contain plant parts that can produce viable offspring and it is thus not regarded as a genetically modified organism. Apart from product release, the extraction procedure also functions as a mechanical pre-pretreatment of the biomass which can accelerate composting as described above. A thermal or chemical inactivation of solids and liquids will only be necessary for processes relying on transient expression mediated by *A. tumefaciens* infiltration into the plants.

### Reduced Ecological Footprint Through Inherent Carbon Dioxide Fixation and Improved Fertilizer Use

Microbial carbon dioxide fixation in fermentation-based systems has recently been proposed as an approach to reduce the ecological footprint of manufacturing in general (Perathoner and Centi, 2014), but this requires substantial investment into infrastructure, e.g., fermenters for microbial cultivation, and is limited by the often slow growth rates of bacteria on C1 carbon sources (Chi et al., 2014; Claassens, 2017) or by difficulties of an establishing suitable metabolism in such organisms (Gong et al., 2016). In contrast, plant-based processes have the potential to function as an immediate and cost-effective carbon dioxide re-fixation tool if operated in close proximity to emission-intensive industries such as steelworks or coal-based power plants. In such cases, the emissions (or even carbon dioxide from air) can be conditioned using filters (Magill, 2017) to ensure sufficient purity and quality, and can then be piped directly to indoor cultivation areas, such as a vertical farm (Wirz et al., 2012). Thereby, the carbon dioxide can used as a gaseous fertilizer (Zhu et al., 2016) for plant cultivation without any potential trade-offs due to nitrogen or water limitation, which can occur in the open field (Reich et al., 2014; Osborne, 2016). This creates a symbiosis between conventional processes and sustainable biotechnology. A similar setup has been reported to work successfully for wheat, if carbon dioxide levels are controlled (Xu, 2015). Implementing such a carbon dioxide fixation into fully automated facilities for the indoor cultivation of plants that have recently been installed and commissioned (Wirz et al., 2012; Holtz et al., 2015) can increase the sustainability of the corresponding processes. Given a typical carbon dioxide fixation rate of 1.0–1.5 g h^-1^ per plant for tobacco (Peterson and Zelitch, 1982; Simkin et al., 2015), an annual output of ∼1,500,000 plants per facility each with an average active carbon dioxide fixation time of 200 h, such a facility can bind about 300,000–450,000 kg y^-1^ of the climate-changing gas. This is equivalent to the carbon dioxide exhaust generated by a 500 MW coal power plant in 1 h (Cebrucean et al., 2014).

The sustainability of indoor cultivation can be further increased by an improved fertilizer utilization. The production of fertilizers is energy-intensive, due in particular to their nitrogen-containing components. The Haber–Bosch process (fixation of molecular nitrogen as ammonia) alone accounts for ∼1.2% of the global energy demand, which is equivalent to 6.5 ZJ (1 ZJ = 1 zeta joule = 10^21^ J) (Gilbert and Thornley, 2010; IEA, 2015). Even so, more than 30% of the fertilizers applied to open fields are inaccessible to the plant, e.g., due to nitrification–denitrification (Aulakh and Bijay, 1996; Seitzinger et al., 2006; Huang et al., 2017). Given fertilizer prices of 0.8 € kg^-1^ and typical fertilization rates of 210–270 kg ha^-1^ y^-1^ (Billen et al., 2013; Li et al., 2013; Van Grinsven et al., 2013; Brown et al., 2014), this represents an economic loss of about 42 € ha^-1^ y^-1^ or 1,500–2,000 € y^-1^ for an average 30-ha farm in developed countries (Lowder et al., 2016). Such a loss corresponds to about 6% of the annual 20,000 € income of an average farmer in the 28 countries of the European Union (Unit Farm Economics, 2018). Furthermore, fertilizers can be washed from the soil into groundwater (Soldatova et al., 2017), not only further decreasing the bioavailable nitrogen but also resulting in the pollution of potable water resources with nitrates, potentially causing diseases such as methemoglobinemia (van Grinsven et al., 2006) or colorectal cancer (Espejo-Herrera et al., 2016) as well as increasing the emission of the greenhouse gas nitrous oxide (Jurado et al., 2017). In contrast, contained cultivation setups such as greenhouses and especially vertical farms have the potential to reduce groundwater contamination as well as water consumption due to closed-loop irrigation systems, which will be beneficial in the context of sustainable agricultural production in the future (Pfister et al., 2011; Roy et al., 2012). If artificial growth supports such as mineral wool are used, fertilizer consumption is also likely to decrease because >95% of the substrates are made available to the plant in this context (Bussell and Mckennie, 2004). Additionally, scenarios for the re-use of artificial growth supports are actively being investigated (Jeong and Hwang, 2001), increasing the sustainability of this approach.

The sustainability of plants will also be a valuable asset if cell-free expression systems are used not only for analytical but also for preparative purposes (Buntru et al., 2014; Harbers, 2014; Zemella et al., 2015), where increasing amounts of cell lysate will be required during process scale-up.

### Using Plant-Bioreactors to Harvest Multiple Products From a Single Process

**FIGURE 3:**
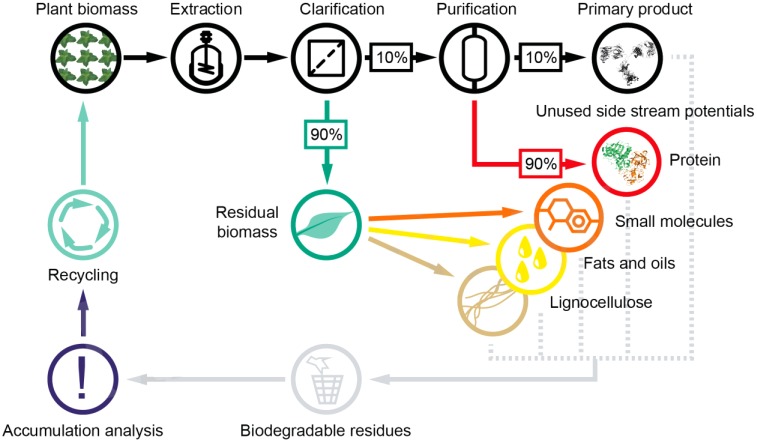
Potential product side streams in plant molecular farming. Most often the primary product constitutes less than 1% of the biomass produced during upstream processing. Opportunities arise for additional revenue when biomass side streams, especially proteins and small molecules, are included in the valorization, which broadens the diversity of the value chain. An integration with existing facilities, e.g., biogas plants, may be required for a cost-effective processing of side products with low margin.

In addition, plants offer a massive window of opportunity for further process integration, i.e., the use of biomass side streams to harvest additional products that can contribute to overall process profitability (Octave and Thomas, 2009). For example, when plants are used to manufacture recombinant proteins, only the product is utilized which often accounts for less than 10% of the total protein content (Figure 3).

Finding applications for the remaining 90% could thus improve process economics. Additionally, plants produce diverse secondary metabolites (Hartmann, 2007; Baranska et al., 2013) which are currently discarded, but these could also be exploited as chemical precursors or even pharmaceuticals (National Research Council [US] Chemical Sciences Roundtable, 2001; Werpy and Petersen, 2004; Erickson et al., 2012; Isikgor and Becer, 2015; Buyel, 2018). Because the extraction conditions for such metabolites often differ from those of proteins (Jones and Kinghorn, 2012; Santos-Buelga et al., 2012; Seidel, 2012; Routray and Orsat, 2013; Buyel, 2015), orthogonal processing by sequential extraction would be possible and could be exploited as an initial purification step. The residual biomass, mostly lipids and lignocellulose, could then be processed to yield further chemical building blocks or biofuels, as has been the focus of extensive research over the last decade (Li et al., 2014; Klose et al., 2015; Roth and Spiess, 2015; Lambertz et al., 2016; Willis et al., 2016). Utilizing these side streams has a low risk because biomass from wild type as well as transgenic plants tends not to contain human pathogens, whereas the waste streams from mammalian cell cultures may contain infectious adventitious viruses (Jennings, 1998; Merten, 2002; Bethencourt, 2009).

#### The Potential of Secondary Protein Products

In plant molecular farming, the target protein often accounts for less than 10% of the TSP (Buyel, 2015), even if expression levels are >2 g kg^-1^ biomass (Bendandi et al., 2010; Zischewski et al., 2015). The remaining TSP fraction comprises HCPs and optionally various fluorescent marker proteins to facilitate expression analysis and accessory proteins that boost product expression, e.g., DsRed and p19, respectively (Arzola et al., 2011; Garabagi et al., 2012; Shamloul et al., 2014; Sack et al., 2015). All these proteins are typically removed during product purification, e.g., by precipitation, filtration, or chromatography, and are discarded as waste (Wilken and Nikolov, 2012; Buyel et al., 2014a, 2015c). The disposal of proteins becomes more counterintuitive the further downstream in the process it occurs because value has been added not only to the product but also to the other proteins when they are separated from any dispersed particulate matter and often (e.g., before and after chromatography) comprise a clear, sterile solution. For example, the clarified extract during the production of a plant-derived mAb contained 0.01 mg L^-1^ of the primary product and also ∼0.25 g L^-1^ of the fluorescent marker protein DsRed (Buyel and Fischer, 2014c). DsRed was used as a visual marker to identify plant lines with high expression levels of both the marker and the primary mAb product (Sack et al., 2015), and was therefore present in the same biomass at no additional expense. We have extracted DsRed from the side stream and achieved a purity of ∼95% using a simple combination of heat treatment, immobilized metal-ion affinity chromatography, and lyophilization with a yield of 80%, corresponding to ∼1.00 g kg^-1^ biomass at a total cost per 200 kg biomass (including labor and consumables) of €2,000.

Assuming the same annual biomass output as above, and market prices of 6,250–6,360 € per 5 mg for DsRed of similar purity [prices retrieved from “Biovision” (#4997-5000) and “mybiosource” (#MBS844904) on July 27, 2017], this is equivalent to an unused economic potential of 6–200 million euros (∼1,250,000 € kg^-1^ biomass) depending on the process scale. It is likely that market prices will drop once large quantities of the fluorescent protein become available, but even if prices decline by a factor of 1,000 compared to the current level, this would still add an extra revenue of 1,250 € kg^-1^ of plant biomass. The market size for such fluorescent proteins may appear small on first sight, i.e., limited to a specialized scientific community, but due to their intense and various colors as well as their natural origin and stability, such recombinant proteins can be replacements for synthetic food colors which is becoming increasingly important in the European Union and the United States (Lehto et al., 2017). Hence, fluorescent proteins may be added to soft drinks or dairy products. Furthermore, technical enzymes or proteins with applications in diagnostics can be interesting side products to co-express in plant-based systems. Examples for such products may be acetylcholine esterase which has been effectively produced in tobacco and required minimal processing (Rosenberg et al., 2015), or horseradish peroxidase produced transiently in *N. benthamiana* (Walwyn et al., 2015).

#### Recovery of Bulk Protein From Plant Extract

The protein side stream is not only suitable for special proteins but may also be used for bulk protein production. For example, the clarified extract mentioned above also contained ∼2.00 g L^-1^ of residual HCPs (Buyel and Fischer, 2014c). Based on the 3:1 buffer-to-biomass ratio (L kg^-1^) used for extraction, and assuming an annual biomass output of 5,000–180,000 kg (100–3,600 kg w^-1^) (Buyel et al., 2017), this is equivalent to an output of 40–1,440 kg of purified plant protein per year that can be obtained from a vertical farm with a 100–2,000 m^2^ cultivation area without any additional production, extraction, and clarification costs. For comparison, this is about 0.5 ppm of the annual world production of non-sterile unprocessed lentil-derived protein (Klostermeyer et al., 2016). Given the market price of 6.5 € kg^-1^ for plant-derived protein (Bomgardner, 2015), this corresponds to an unused economic potential of up to 10,000 € (or ∼0.06 € kg^-1^ biomass) which will increase with the process scale. Of course, further purification may be required to separate the proteins from host DNA, pigments, and any residual primary product, slightly diminishing the potential revenue. In addition to economic benefits, using side stream proteins can help to reduce the land area, fresh water, and fossil energy required for food production, which in the United States represent 50, 80, and 17% of the total available resources, respectively (Pimentel and Pimentel, 2003), as well as ensuring a weather-independent supply of food if vertical farms are used for production. The latter may also help to reduce food price volatility (Gilbert and Morgan, 2010).

#### Isolating Secondary Metabolites From Plants by Orthogonal Extraction

As well as proteins, plants contain a vast number of secondary metabolites with various applications, e.g., as anti-cancer agents, dyes, or fine chemicals (Staniek et al., 2013; Benedito and Modolo, 2014; Martim, 2014; Cuellar-Bermudez et al., 2015; Oertel et al., 2017; Buyel, 2018). There are two major opportunities to isolate such metabolites from side streams during the production of recombinant proteins: purification from the primary aqueous extract once the target protein has been captured, and re-extraction from residual biomass using solvents that have orthogonal solubilization properties compared to the primary buffer, i.e., organic liquids such as methanol or ethanol (Jones and Kinghorn, 2012; Santos-Buelga et al., 2012; Seidel, 2012). For example, tobacco, which is often used for molecular farming (Stoger et al., 2002; Spiegel et al., 2018), contains a number of different flavonoids and alkaloids, most prominently nicotine (Dueckershoff et al., 2005; Mungur et al., 2005; Buyel et al., 2015a), which are sparingly soluble in the aqueous solutions typically used for protein extraction (Buyel et al., 2015c). However, these small molecules can be released from plant biomass by re-extraction using alcohols, e.g., 40% [v/v] methanol in water acidified with 0.5% [v/v] acetic acid (Keinanen et al., 2001). Among tobacco metabolites, rutin has potential applications in breast cancer therapy (Iriti et al., 2017). Recently, rutin was isolated using a non-optimized procedure from tobacco leaves yielding 0.2–0.7 g kg^-1^ biomass (Keinanen et al., 2001; Buyel et al., 2015a). Using the annual biomass output of 5,000–180,000 kg (100–3,600 kg w^-1^) for a vertical farm (Buyel et al., 2017) along with the current sales prices for rutin of 1.0–1.5 € g^-1^,^2,3^this corresponds to an economic value of 1,000–190,000 € per year or 0.84 € kg^-1^ biomass. The actual margin would be lower due to the additional investments and consumables costs, e.g., the installation of extractors compatible with organic solvents, the cost of the solvents themselves, and the cost of recycling (Jones and Kinghorn, 2012). However, the profit could nevertheless be increased by enhancing the concentration of rutin and similar compounds at least by a factor of five through the selection and optimization of specific lighting regimes and/or the expression of different effector proteins, e.g., from *Pseudomonas syringae* (Buyel et al., 2015a). However, if such modifications are introduced it is important to ensure that they do not compromise the quantity or quality of the primary product as this would create a trade-off between primary and secondary uses that would affect the overall process economics.

#### Chemical Building Blocks Derived From Plant Biomass Side Streams

The largest fraction of biomass accumulated during the plant-based production of recombinant proteins is lignocellulose, accounting for 60–70% of the total dry biomass of tobacco plants (Sheen, 1983). Lignocellulose is a heterogeneous substance composed of cellulose, hemicellulose, and complex lignins, typically in a 1.6:1.0:1.0 ratio (Sawatdeenarunat et al., 2015) or specifically for tobacco in a 1.9:1:1.3 ratio (Canam et al., 2006). Due to this heterogeneity and despite extensive research (Isikgor and Becer, 2015; Rinaldi et al., 2016; Willis et al., 2016; Amin et al., 2017; Bhatia et al., 2017), the effective utilization of lignocellulose to produce fine chemicals and biofuels has been challenging. For example, maintaining a stable multi-organism process (Sawatdeenarunat et al., 2015) is a complex task which can increase the likelihood of failure. Also, harsh process conditions such as low pH (<2.0), high temperatures (>150°C) (Kumar et al., 2009), or large quantities of enzymes (>30 g kg^-1^ cellulose) (Tu and Saddler, 2010; Weiss et al., 2013) may be necessary, which can have a negative impact on the process economics. However, lignocellulose has been recognized as an alternative non-fossil substrate for the chemical industry (Busch et al., 2006; Sanders et al., 2007; Arevalo-Gallegos et al., 2017) and microorganisms may be useful to facilitate processing (Gall et al., 2017) and to produce biobased chemicals (Yenkie et al., 2016). More importantly, recent developments highlight the potential to express specific enzyme sets consisting of exoglucanases, endoglucanases, and β-glycosidases directly within the plant biomass (Jung et al., 2012; Garvey et al., 2013; Lambertz et al., 2014; Park et al., 2016) and activating them at moderate temperatures (∼55°C) (Klose et al., 2013) which can catalyze the breakdown of the large polymers into fragments *in planta*, facilitating their subsequent reduction to chemical building blocks such as benzene, phenol, toluene, or xylene (Rinaldi et al., 2016). Of course, the expression of such additional proteins will require validation to ensure that primary product expression is not affected in terms of quality and quantity. Based on a reported effectiveness for phenol release from lignin of 0.5 kg kg^-1^ (Rinaldi et al., 2016), in combination with a lignin yield of 0.02 kg kg^-1^ of wet tobacco plant biomass (Canam et al., 2006; Buyel, 2016) and typical processing costs of 0.02 € kg^-1^ dry biomass as well as selling prices of 1.20 € kg^-1^ for phenol (Rinaldi et al., 2016) this has the potential for additional valorization of 0.01 € kg^-1^ biomass or 50–1,800 € per year for a typical vertical farm. Also, the residual biomass from plant-derived biopharmaceutical production may be used to manufacture the biobased and biodegradable plastics mentioned above (see Sustainability of plant-based expression), such as regenerated cellulose (Rujnic-Sokele and Pilipovic, 2017), for which market growth of up to 400% has been predicted until 2019 (Prieto, 2016). Alternatively, plant waste may be subjected to further microbial processing to yield high-value products as shown for the transformation of nicotine from tobacco waste into 6-hydroxy-3-succinoyl-pyridine (Yu et al., 2014). Plant waste may also have applications in the automotive industry as precursors for carbon fibers (Mainka et al., 2015).

#### The Last Resort: Bioenergy and Biofuel Production

Even if the directed processing of residuals is not feasible, i.e., if specific compounds cannot be extracted or generated, it is still possible to use the remaining plant biomass as feed for microbial processes to produce polymers and bioenergy (Bhatia et al., 2017; Pagliano et al., 2017) or for biogas production in well-established composting devices (Yuan et al., 2008). For such devices to work properly, mechanical pre-treatment may be required to increase the surface area on which microbial degradation of the biomass can take place (Amin et al., 2017; Rodriguez et al., 2017). These surfaces will automatically be generated if substrates are derived from molecular farming applications because the plant material is often homogenized for the purpose of product extraction (Buyel et al., 2015c) yielding a particle size distribution in the 0.5–10.0 μm range (Buyel and Fischer, 2014b). Using such pre-processed substrates can simplify the biomass conditioning and thus reduce costs.

One can anticipate a typical methane output from anaerobic digestion of lignocellulose biomass of 340 ± 50 Nm^3^ m^-3^_V S._ (cubic meters of gas under standard conditions, i.e., 1,013.25 Pa and 273.15 K, per cubic meter of volatile solids/organic matter) (Weiland, 2010). Based on an annual vertical farm output of 5,000–180,000 kg (100–3,600 kg w^-1^) biomass (Buyel et al., 2017) with a dry mass content of about 0.1 kg kg^-1^ (Buyel, 2016), a share of 90% volatile solids (Sheen, 1983) and assuming a density of 1.0 kg L^-1^ dry mass, this corresponds to a methane production capacity of 130–6,300 Nm^3^ per year depending on the size of the vertical farm and the efficacy of anaerobic digestion, or about 0.035 Nm^3^ methane per kg wet biomass. With a density of methane of 0.717 kg m^-3^, a lower heating value of methane of 37.5 MJ kg^-1^, and assuming a biogas recovery of 0.75 and a gas engine efficiency of 0.33 (Surroop and Mohee, 2011), this produces 1,700–81,700 MJ per year and vertical farm, or 0.33–0.45 MJ kg^-1^ biomass. This energy can be directly re-fed into the vertical farm to reduce the energy demand, e.g., for illumination and climate control, reducing the associated costs. Taking energy prices of 0.024 € MJ^-1^ (Boulamanti and Moya, 2017) into account, this yields an extra revenue of 0.008–0.010 € kg^-1^ biomass or 40–2,000 € per year and facility. Alternatively, methane may be sold directly as a C1 source for fermentation or synthesis at a price of ∼0.30 € kg^-1^ (Boulamanti and Moya, 2017) corresponding to ∼0.006 € kg^-1^ biomass. It should also be taken into account that bioenergy produced by such an integrated process will not compete with food and feed production, which has been a concern for biofuels derived from maize and other staple plants (Popp et al., 2014).

#### The Economy of Scale

With the exception of recombinant protein side products, the additional revenues generated by the process integration steps described above are apparently low (i.e., <1.0 € kg^-1^ biomass), especially when traded against the costs for the installation of the necessary devices. The low margins may thus not justify the installation of biogas facilities or other side processes from an economical perspective, even for large-scale operations that have recently been outlined for the production of mAbs in plants (Buyel et al., 2017). The low margins will also be prohibitive for shipping the residual biomass to existing facilities for further processing. It will thus be necessary to integrate plant-based processes beyond the side product level in order to fully exploit the potential of the produced biomass. For example, large-scale vertical farm units for the production of recombinant proteins can be located directly next to existing biogas facilities, e.g., around farms or wastewater plants. Thereby, transportation costs can be minimized or even avoided and the capacity utilization of the facilities used for biomass processing can be increased. In this context, the small footprint and independence of climate conditions will be an important asset for plant-based recombinant protein expression in vertical farms because they grant a high degree of flexibility for the individual design of a process integration.

### The Impact of the Socio-political Framework for Plant Molecular Farming on Process Integration

The socio-political situation, including consumer acceptance, for genetically modified plants is diverse from a regional, a product-based, and an individual perspective and its analysis is beyond the scope of this study. However, we will briefly discuss the aspects that are relevant for the techno-economic part of a process integration scenario using genetically modified plants, i.e., process safety and social acceptance as a measure for a future product turnover.

Acceptance of gene technology, especially when applied to plants for food and feed, is on average higher in the United States than in the EU. The political climate and a reduced relevance of the technology for crops typically cultivated in the EU compared to the United States have been identified as potential reasons for this situation (Zilberman et al., 2013). Whereas more than 30% of the EU farmers are still interested in cultivating genetically modified plants due to profit increases of up to 68% (Lucht, 2015), consumers have reservations because the technology is often advocated by distrusted multinational corporations (Arntzen et al., 2003) and the capacity of regulatory authorities to ensure product and environmental safety is doubted due mosaicism in legislation (Ishii and Araki, 2016). Interestingly, these reservations mostly apply to foods but not medicines derived from processes using gene technology, which has been partially attributed to a biased coverage by the media (Zilberman et al., 2013). A multi-national field trial on the other hand showed that in an actual head-to-head comparison (organic, conventional, and genetically modified product) more than 20% of the consumers selected the genetically modified food product, especially when a reduced price reflecting the potential cost savings in production was included (Knight et al., 2007). However, taking the latest developments in labeling of cisgenic plants (van Hove and Gillund, 2017) and the assessment of CRISPR/Cas as a gene technology into account (Callaway, 2018), focusing on technical (the section “The potential of secondary protein products”) rather than food- or feed-based side products (the section “Recovery of bulk protein from plant extract”) seems beneficial at the moment if the primary product relies on gene technology.

Public acceptance can also affect process safety, for example if cultivation areas for genetically modified plants are destroyed by environmental activists (Nausch et al., 2015). Transferring the production into vertical farms could improve the situation in two directions because the fully enclosed facilities minimize both, the chance for unauthorized access to the production and the likelihood of an uncontrolled spread of genetically modified plant (seeds) into the environment.

## A Case Study for an Integrated Plant-Based Process

**FIGURE 4:**
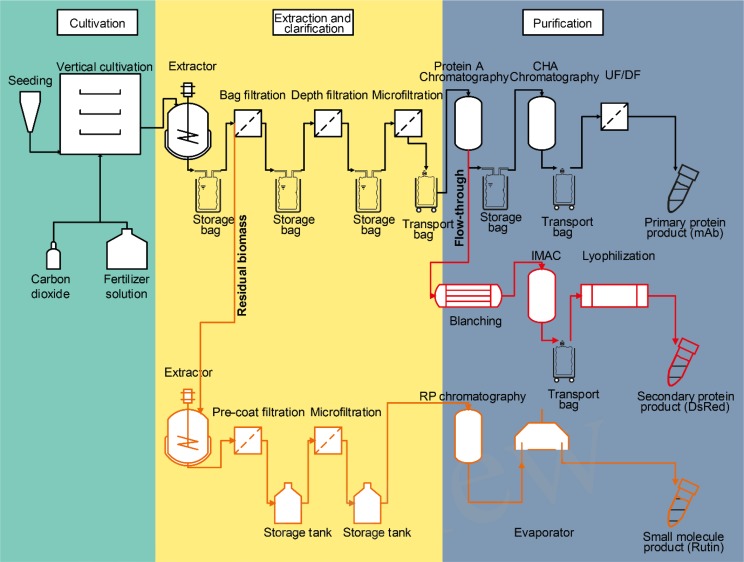
Flow scheme of a partially integrated plant-based production process. The primary process (black) produces a high value pharmaceutical product, here, a mAb. Several side streams (bold) can be used to extract additional products. For example, a re-extraction of the residual biomass (orange) with an organic solvent can yield different small molecule products like rutin. Furthermore, co-expressed technical enzymes or diagnostic agents like DsRed can be isolated from liquid process wastes like the flow-through of a chromatography step (red). Each of the process branches stretch through one or several of the three typical process phases, i.e., plant cultivation (green background), extraction, and clarification (yellow background) and purification (blue background). CHA, ceramic hydroxyl apatite; IMAC, immobilized metal-ion affinity chromatography; RP, reversed phase; UF/DF, ultrafiltration/diafiltration.

As highlighted in the section “Developing the Potential of Plant-Based Production Systems,” there are several options to derive additional products from process side streams and to use the latter to increase the overall process profit margin. In this section, we will investigate the effect of integrating two side products, i.e., an additional protein and one small molecule, on the overall process economics for the production of a recombinant mAb in plants. Due to cost constraints arising from the scale (see section “The Economy of Scale”), biogas, and chemical building block production from residual biomass are not covered here but will be part of an upcoming case study. Based on a process already established in our facility at the Fraunhofer IME in Aachen, Germany (Figure 4), the costs of goods and labor for the production of 77 g of antibody from 200 kg of transgenic tobacco (*N. tabacum*) biomass from a single batch, of which 12 can be performed per year, accounted for ∼59,000 € per batch corresponding to ∼750 € g^-1^ of purified product or ∼200 € kg^-1^ biomass (Table 1). These costs of goods for the primary process depend on several boundary conditions such as the expression level, which was 0.48 g kg^-1^ in this case. The costs covered all consumables (including chromatography resins, energy, and disposal costs as well as quality control and quality assurance along with maintenance), chemicals, and labor but did not account for depreciation and taxes. Assuming low market prices of ∼2,000 € g^-1^ purified antibody (Kelley, 2009), the revenue of the plant-based process would be 154,000 € or ∼770 € kg^-1^ biomass for the mAb alone.

**Table 1:** Economic potential of process integration for plant-derived products.

Factor	Type	Economic impact (€ kg^-1^ biomass)	Reference
Cultivation	Input	-150	Buyel and Fischer, 2012
Aqueous extraction	Input	-40	Buyel and Fischer, 2012; Buyel and Fischer, 2014a
Product purification	Input	-42	Buyel and Fischer, 2012, 2014c
Primary protein product	Output	500–1,200^1^	Kelley, 2009; Buyel and Fischer, 2012
Protein purification	Input	-10	Buyel and Fischer, 2012
Secondary protein product	Output	50–1,000^1^	This study
Residual protein	Output	0.05–0.25^1^	Bomgardner, 2015
Organic extraction	Input	-0.5	This study
Small molecule purification	Input	-0.5	This study
Small molecule product	Output	0.9–2.0^1^	This study
Sum	Total	308–1104	–


*^*1*^Values depend on the type and expression level of primary and side-product.*

Integrating the purification of DsRed from the Protein A flow-through stream added costs of about 2,000 € for purification at the 200 kg scale (including labor and consumables) but at the same time this provides additional revenue of ∼1,000 € kg^-1^ biomass assuming an expression level of 1.0 g kg^-1^ biomass, a recovery of 80% and that the fluorescent protein can be sold for at least 0.1% of the prices currently posted by large providers of chemicals and reagents (∼1,250 € mg^-1^, prices retrieved as above).

If the small molecule rutin can be obtained by re-extraction of the residual biomass (see Section “Isolating Secondary Metabolites From Plants by Orthogonal Extraction”), yielding 0.7 g kg^-1^ biomass, an additional revenue of 0.84 € kg^-1^ biomass can be generated with estimated costs of 0.5 € kg^-1^ biomass to account for labor and consumables, including solvents (here an 80:1:99 methanol:acetic acid:water mixture), filters and a one-step chromatographic purification using preparative reversed-phase HPLC assuming a resin life-time of 50 cycles. Because the margin for rutin is small, future work should focus on a more detailed analysis of the actual life time of the necessary process equipment like the reversed-phase resin, as this will greatly affect the economic viability of the process.

Therefore, exploiting the potential of several side streams has the capacity to more than double the revenue of plant based-processes, e.g., from ∼770 to ∼1,770 € kg^-1^ biomass in the case of a mAb. From the data presented here, it is clear that additional recombinant proteins as well as secondary metabolites have the highest direct economic benefit if produced in an integrated approach (Table 1 and Supplementary Table S1). However, increasing energy prices and more stringent environmental protection legislation in the future may favor the incorporation of full biomass utilization through biogas and energy production as well as using plant-based production for direct carbon fixation. Additionally, the entire process will benefit from an increasing production scale and there is a potential to use biomass from plant-based processes in nearby facilities like biogas plants often located at agricultural sites to increase facility utilization and revenue.

## Conclusion

Plants offer multiple advantages for the production of recombinant proteins but their adoption by industry is hindered by the fact that mammalian and other cell cultures are much more established and better characterized in an industrial setting, making it hard for plant-based processes to gain a foothold in the market. Therefore, the additional potential of plant-based systems may need to be exploited to tip the balance in favor of sustainable plant-derived products. Here we have shown that, in theory, integrating the products from various side streams can more than double the process revenue, increasing the economic competitiveness of the corresponding products accordingly, especially when protein side products are produced. Furthermore, using plants as a sustainable production platform may avoid penalties by regulatory authorities in the future because such platforms better match the goals of environmental protection legislation. Furthermore, using plant-based expression systems has the potential to reduce the dependence on oil-based products as has been reported for improved manufacturing techniques in other sectors (Kagawa et al., 2009), which can increase the robustness of production in the face of oil price fluctuations.

In the future, it will be important to show that the improvements described above can be implemented and scaled into an actual production process with relevant side products, which has not yet been achieved for non-protein side streams. In this respect, identifying novel mid-to-high value side products that can be derived cost-effectively from the residual biomass will be important for additional increases in process revenue. Further research should focus on the integration of the steps outlined above in an enclosed plant-based production facility such as a vertical farm in combination with established bio-refineries. This will allow for additional process intensification due to the increased space time yield (volumetric productivity) of such units compared to cultivation in the open field or in a greenhouse. A thorough comparative analysis of the three major types of plant cultivation (open-field, greenhouse, and vertical farm) should be carried out to determine the relative impact of energy and fertilizer consumption, logistics, and socio-political acceptance, in order to fine-tune future implementations and achieve success. If the recirculation of process streams is considered, e.g., using the remains of biogas production as fertilizers for another round of plant growth, a detailed risk analysis in terms of the accumulation of toxic compounds will also be required.

## Author Contributions

JB has analyzed the data and literature and written the manuscript.

## Conflict of Interest Statement

The author declares that the research was conducted in the absence of any commercial or financial relationships that could be construed as a potential conflict of interest.

## Footnotes

1 https://urbancropsolutions.com/

2 www.sigmaaldrich.com

3 www.carlroth.com

## Abbreviations

DSP: downstream processing
HCP: host cell protein
mAbs: monoclonal antibodies
TSP: total soluble protein
