# Supplementary material for: Plant Molecular Farming – Integration and Exploitation of Side Streams to Achieve Sustainable Biomanufacturing
Source: Front Plant Sci. 2019 Jan 18;9:1893. doi: 10.3389/fpls.2018.01893 (PMC6345721; doi:10.3389/fpls.2018.01893)
Supplement: Supplementary file 1 [file Table_1.pdf]

Supplementary materials for:

Title: Plant molecular farming – integration and exploitation of side streams to achieve sustainable biomanufacturing

J. F. Buyel[1,2]

Supplementary data

Supplementary table S1: Possible process side streams for transgenic *Nicotiana tabacum*-based biopharmaceutical protein production. References for details on content and prices are provided in the text.

Process stream	Substance	Content [g kg^{-1} biomass]	Price [€ kg^{-1}]	Revenue [€ kg^{-1} biomass][1]	Absolute revenue per 200 kg batch [€]
Primary protein product	mAb M12	0.48	2,000,000	770	154,000
Secondary protein product	DsRed	1.00	1,250,000[3]	1250	250,000
Small molecule product	Rutin	0.70	1500	1	200
Residual bulk protein[2]	Protein mixture	6.52	6.50	0.03	8.48
Lignocellulose (chemical building block)[2]	Phenol	10.00	0.95	0.01	1.90
Lignocellulose (biogas)[2]	Methane	25.10	0.30	0.01	1.51
Revenue for integrated process[4]				1771 (1771.1)	403,200 (403,212)
Surplus revenue from process integration[4]				1001 (1001.1)	250,200 (250,212)

[1] A recovery of 0.8 [-] was assumed for all process streams.

[2] These side products have not been included in the case study presented here.

[3] The price depends on the nature of the secondary protein product and may be below 10,000 € kg^{-1} product for technical or diagnostic products. Accordingly, the revenue would be 10 € kg^{-1} biomass and 2000 € per 200 kg batch.

[4] Numbers indicate the values for the process presented in the case study, whereas numbers in brackets indicate the values for a total integration using all process streams.
